# Enzymes involved in trehalose-chitin synthesis in *Haemonchus contortus* could be vaccine candidates for goats

**DOI:** 10.1186/s13071-025-06703-4

**Published:** 2025-02-20

**Authors:** Zhaohai Wen, Jilata Amu, Kalibixiati Aimulajiang, Jiajun Feng, Cheng Chen, Yongde Xu, Mingmin Lu, Lixin Xu, Xiaokai Song, Xiangrui Li, Ruofeng Yan

**Affiliations:** 1 MOE Joint International Research Laboratory of Animal Health and Food Safety, College of Veterinary Medicine, Nanjing Agricultural University, Nanjing, 210095 Jiangsu People’s Republic of China; 2 Department of Veterinary Medicine, College of Coastal Agricultural Sciences, Guangdong Ocean University, Zhanjiang, People’s Republic of China; 3 State Key Laboratory of Pathogenesis, Prevention and Treatment of High Incidence Diseases in Central Asia, Xinjiang Medical University, Urumqi, 830011 Xinjiang People’s Republic of China

**Keywords:** *Haemonchus contortus*, HcTPS, HcGOB, Vaccine, Trehalose-chitin synthesis

## Abstract

**Background:**

Trehalose-6-phosphate synthase (HcTPS) and trehalose-6-phosphate phosphatase (HcGOB) are key enzymes for trehalose synthesis in *Haemonchus contortus*. In addition, previous studies have also demonstrated that HcTPS and HcGOB can regulate the function of host immune cells in vitro, and are important immunosuppressive molecules. Therefore, this study evaluated the potential of HcTPS and HcGOB as vaccine candidates through in vitro and in vivo experiments.

**Methods:**

To evaluate the inhibitory effects of polyclonal antibodies on egg hatching and larval development, anti-rHcTPS and anti-rHcGOB antibodies were incubated separately with eggs and first-stage larvae (L1s) under controlled in vitro conditions. For immunization studies, recombinant proteins (rHcTPS and rHcGOB) were formulated with Quil-A adjuvant, and administered to goats through subcutaneous injection. Vaccine efficacy against *Haemonchus contortus* infection was determined through comprehensive analysis of multiple parasitological parameters, including: (1) egg abnormality rate, (2) hatching success rate, (3) reduction egg output rates, and (4) reduction in adult worm burden.

**Results:**

The results of in vitro experiments showed that polyclonal antibodies against HcTPS and HcGOB had no effect on the hatching rate of eggs, but significantly affected the development from L1s to infectious third stage larvae (L3s). After immunization with recombinant HcTPS protein (rHcTPS) and recombinant HcGOB protein (rHcGOB), high levels of antigen-specific immunoglobulin G (IgG) were produced in goats, and remained till the end of the experiment. Compared with the Quil-A adjuvant control group, the number of deformed eggs in the rHcTPS protein- immunized group and the rHcGOB protein- immunized group were significantly increased. In the rHcTPS protein-immunized group and the rHcGOB protein-immunized group, the deformity rate of eggs was 9.59% and 17.30%, respectively, and the hatching rate of eggs was reduced by 11.27% and 13.71%, respectively. Moreover, compared with the Quil-A adjuvant control group, the number of eggs and adults in the HcTPS protein- immunized group decreased by 64.47% and 60.93%, respectively, and the number of eggs and adults in the rHcGOB protein- immunized group decreased by 63.97% and 69.54%, respectively. Furthermore, compared with the control group (Quil-A adjuvant), the trehalose content in the rHcTPS protein- immunized group and the rHcGOB protein- immunized group was also significantly reduced.

**Conclusions:**

These findings indicate that rHcTPS and rHcGOB exhibit superior immune protective effects, rendering them promising candidates for vaccine development.

**Graphical Abstract:**

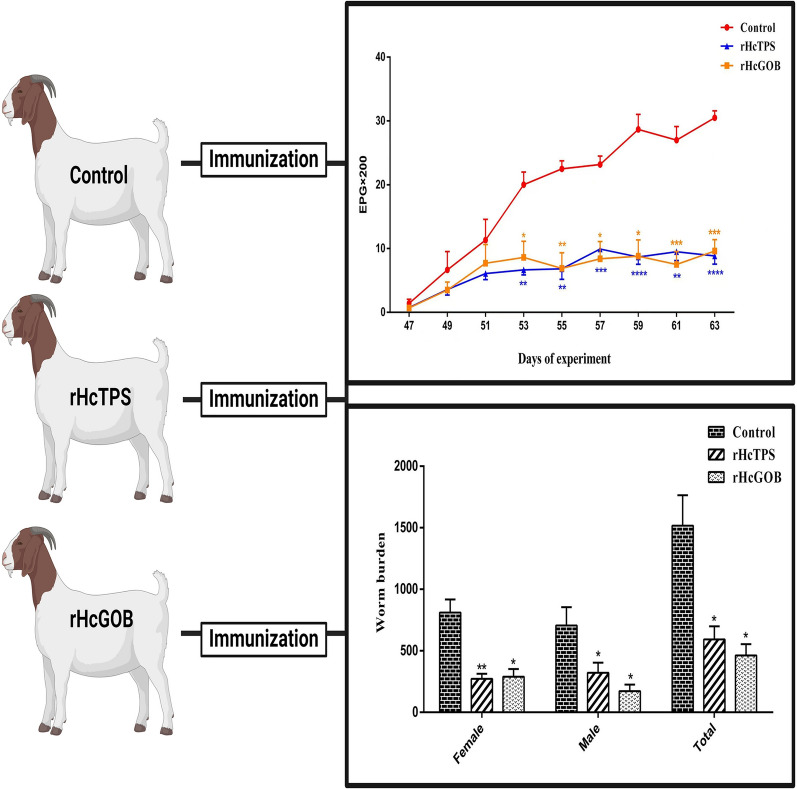

## Background

The severe infection of *H*. *contortus* poses a significant threat to the health of ruminant animals, such as goat and sheep, resulting in substantial economic losses for the global livestock industry and negative impacts on animal welfare [1–4]. Infection with *H*. *contortus* can result in emaciation, anemia, and other clinical manifestations in small ruminants, and may lead to the mortality of affected animals when severe, particularly posing a significant threat to lambs [5, 6]. Currently, the main types of anthelmintic drugs for livestock include benzimidazole, macrocyclic lactone, and imidazothiazole [7–9]. Excessive utilization of chemical deworming agents, however, not only contributes to the development of drug resistance in parasites [7, 10, 11] but also presents a significant risk to public health and safety. Therefore, it is urgent to find new alternative solutions for the prevention and control of hemonchosis.

Vaccination is considered one of the most promising strategies for preventing and controlling *H*. *contortus*; a blood-sucking nematode disease in ruminant animals. Currently, there is only one commercially available vaccine (Barbervax^®^) for the prevention and control of *H*. *contortus* [12]. However, the antigens of the Barbervax^®^ vaccine (H11 and H-gal-GP) need to be extracted from adult worms, which is expensive and difficult to promote comprehensively. Recent studies have reported that antigen molecules derived from the excretory and secretory proteins of *H*. *contortus* showed certain efficacy against *H*. *contortus* infection [13, 14]. However, compared with commercial Barbervax^®^ vaccines, the protective effects of subunit vaccines against *H*. *contortus* infection are still far from satisfactory. Therefore, there is an urgent need to mine new vaccine-candidate molecules.

Trehalose-6-phosphate phosphatase (HcGOB) and trehalose-6-phosphate synthase (HcTPS) are two key enzymes of trehalose synthesis in *H*. *contortus*, and they are expressed in different developmental stages of *H*. *contortus*. Trehalose is widely recognized for its ability to confer protection on organisms and enhance their resilience in challenging environments [15–17]. Simultaneously, trehalose serves as the precursor for chitin synthesis in invertebrates [18]. It is noteworthy that the trehalose chitin biosynthesis pathway is absent in mammals, thus rendering the key enzyme molecules of this pathway as potential targets for anthelmintic drug development [19, 20]. Prior research has demonstrated that the inhibition or deletion of trehalose-6-phosphate synthase and trehalose-6-phosphate phosphatase functions disrupt the trehalose-chitin synthesis pathway, leading to significant repercussions on insect growth, development, and reproduction [17, 21–24]. However, the roles of HcTPS and HcGOB in the growth, development, and reproduction of *H*. *contortus* remain poorly understood.

Our previous study found that HcGOB and HcTPS were mainly distributed in the intestinal microvilli of *H*. *contortus* adults [25, 26], which was consistent with the localization of Barbervax vaccine antigens (H11 and H-gal-GP) in adults [27]. Moreover, both rHcTPS protein and rHcGOB protein can bind to goat peripheral blood mononuclear cells (PBMCs) in vitro and inhibit the immune function of the host. The above results indicate that HcTPS and HcGOB may be potential vaccine antigens.

In this study, the potential of rHcTPS protein and rHcGOB protein as a vaccine candidate molecules was analyzed by in vivo and in vitro experiments, providing a new idea for the prevention and control hemonchosis.

## Methods

### Ethics approval and consent to participate

This study was approved by the Ethics Committee for Animal Welfare and Ethics of Nanjing Agricultural University (NJAU, no. 20221212234), and followed relevant regulations on national experimental animal welfare ethics.

### Parasites and animals

*H*. *contortus* was isolated in Nanjing and reproduced in goats that were free of worms, as mentioned previously [28]. In addition, the culture and collection of infectious third-stage larvae of *H*. *contortus* (L3s) was done as described in previous studies [29, 30]. Healthy goats, aged 3–6 months, were procured from a local family-operated farm in Lai’an County, Chuzhou City, Anhui Province. The goats exhibited uniform body size and nutritional status, with an average weight of approximately 20 kg. After confirming that there was no gastrointestinal parasite infection through fecal examination [31] (No worm eggs and larvae were observed in fecal samples by the floating method and precipitation method), the goats were housed in the general animal room of the Experimental Animal Center at Nanjing Agricultural University’s College of Veterinary Medicine. They were fed alfalfa pellets, shelled corn kernels, and hay daily, and had free access to drink water provided by automatic waterers.

### Preparation of recombinant proteins and production of polyclonal antibodies

The recombinant plasmids (pET28a/HcTPS and pET28a/HcGOB) were introduced into *Escherichia coli* BL21 (DE3), followed by induction of rHcTPS protein and rHcGOB protein expression using isopropyl-d-thiogalactoside (IPTG). The rHcTPS protein and HcGOB protein were purified using Ni^2+^–nitrilotriacetic acid (Ni–NTA) column (Merck, Darmstadt, Germany) in accordance with the manufacturer’s protocols. The rHcTPS and rHcGOB proteins were successfully purified with high efficiency. For detailed results, refer to our previous studies [25, 26]. Meanwhile, the preparation of polyclonal antibodies against rHcTPS and rHCGOB proteins is described in previous studies [25, 26].

### Analysis of the hatching rate of eggs and the development rate of larvae

The approaches for egg hatching and larval culture were founded on previous studies and have undergone minor improvements [32, 33]. The freshly excreted fecal samples were obtained from the rectum of goats harboring *H*. *contortus*, subsequently homogenized, and diluted with an appropriate volume of deionized water. Subsequently, the fecal samples were filtered through an 80-mesh copper sieve to collect the filtrate. The eggs were precipitated by centrifugation at a speed of 1750 rpm for 10 min, followed by removal of the supernatant. Next, 100–150 mL of saturated salt water was added and vortexed for mixing before being centrifuged at a speed of 2000 rpm for another 10 min. The resulting supernatant was collected while the filtrate was passed through a cell sieve with a pore size of 40 µm to remove any remaining saturated salt water. Finally, the eggs on the cell screen were washed down with deionized water to obtain a pure egg suspension. Each well in a culture plate containing 48 wells received a suspension consisting of approximately 1 mL eggs (around 100 per well). Different dilution ratios (1:10, 1:40, 1:160, 1:640, and 1:2560) of rat serum anti-recombinant protein (rHcTPS or rHcGOB) or normal rat serum were added into each well, while benzimidazole fungicide (at a concentration level of 1 μg/mL) served as control group. The cell culture plate was incubated in a biochemical incubator at 28 ℃ for 24 h, and egg hatching was counted. The calculation formula is: hatching rate of eggs = L1s/ (L1s + unhatched eggs) × 100%.

The 48-well cell culture plates were each supplemented with 1 mL of L1s suspension (approximately 100 L1s/well). Subsequently, rat serum anti-recombinant protein (rHcTPS or rHcGOB) or normal rat serum were added at varying dilution ratios (serum dilution ratios: 1:10, 1:40, 1:80, 1:160, 1:320, 1:640, 1:1280, and 1:2560), while albendazole (at a concentration of 1 μg/mL) was included as the positive control group. Simultaneously, each well received an addition of *E*. *coli* solution (OD_600_ = 0.5) in a volume of 10 μL before being incubated at a temperature of 28 ℃ for a duration of 120 h. Finally, the developmental rate of L3s was calculated. Calculation formula: the developmental rate of L3s = L3s/total number of larvae × 100%.

### Vaccination design and animal grouping

All goats were housed in the general animal facility of Nanjing Agricultural University’s Experimental Animal Center for approximately 20 days, during which rectal fecal samples were collected every 5 days to ensure the absence of gastrointestinal parasitic infections prior to the commencement of the experiment. Goats were randomly divided into three groups, group A (Quil-A adjuvant control group), group B (rHcTPS protein immunization group), and group C (rHcGOB protein immunization group), with six goats in each group, half male and half female. On day 0 of the experiment, group A (the control group) received a subcutaneous injection of a mixture containing 500 µg Quil-A adjuvant and sterile water, with a total volume of 2 mL per goat. Each goat in group B was immunized subcutaneously with a mixture comprising 500 µg rHc-TPS protein and 500 µg Quil-A adjuvant, also with a total volume of 2 mL per goat. Group C goats were immunized subcutaneously with a mixture comprising 500 µg rHcGOB protein and 500 µg Quil-A adjuvant, also with a total volume of 2 mL per goat. After a period of 14 days following the initial administration, the second dose was administered using the same dosage and schedule as the first. On the 14th day post-secondary immunization, all experimental goats were infected with 8000 L3s through oral ingestion. On day 64 (35 days after infection), autopsy examination was conducted on the goats.

### Detection of specific IgG in goat serum

On days 0, 14, 28, 35, 42, 49, 56, and 63 of the experiment, the serum samples of each goat were collected by negative pressure blood collection tubes and placed in the −30 ℃ refrigerator for storage.

Checkerboard titration was used to determine the optimal coating amounts of rHcTPS protein and rHcGOB protein, which were 100 ng/ well and 500 ng/ well, respectively, and the optimal dilution ratio of serum was 1:200. Detection of specific immunoglobulin G (IgG) antibody levels in goat serum by enzyme-linked immunosorbent assay (ELISA) was as described previously [34]. IgG antibodies against recombinant protein (rHcTPS or rHcGOB) in serum and abomasal mucosa were detected by ELISA. The recombinant protein was diluted to the appropriate concentration with 0.05 M carbonate coating solution, added to the ELISA plate at 100 µL per well, and coated overnight in a 4 °C refrigerator. After discarding the coating solution, 200 µL of phosphate buffered saline with Tween 20 (PBST) was added to each well and washed three times for 5 min each time. Blocking solution [3% bovine serum albumin (BSA) solution, prepared in PBST] was added to the ELISA plate, 100 µL/well, and incubated in a 37 ℃ incubator for 1 h. After discarding the blocking solution, 200 µL of PBST was added to each well and washed three times for 5 min each time. Next, 100 µL diluted serum (1:200 dilution) was added to each well of the ELISA plate and then incubated in a 37 ℃ incubator for 1 h. After PBST cleaning, 100 µL HRP-conjugated rabbit anti-goat IgG (1:5000, diluted in PBST) was added to each well and incubated in a 37 ℃ incubator for 1 h. After PBST cleaning, 100 µL of TMB color-developing solution was added to each well, and reacted at room temperature in the dark for 15 min. Subsequently, 50 µL of termination solution (2 M H_2_SO_4_) was added to each well and placed in a full wavelength microplate reader to determine the absorbance value at OD_450_.

### Detection of specific IgG in abomasal mucosa

A sample of 100 mg of the abomasal mucosa was weighed, and 1 mL of pre-cooled PBS solution was added. Subsequently, it was placed in a tissue homogenizer for thorough homogenization. The supernatant was collected by centrifugation at 12,000 rpm for 10 min at 4 °C. The supernatant protein concentration was determined by the bicinchoninic acid (BCA) method, and each sample was adjusted to a uniform concentration. The specific IgG level in the homogenate supernatant of abomasal mucosa was detected by ELISA. The specific operation steps are the same as for goat serum, described above.

### Egg hatching rate in different immunization groups

The collection of eggs and the calculation of hatching rate are described above. The eggs were diluted to a concentration of 100–150 per mL with deionized water and added to the 48 wells in the cell culture plate. The plate was then be placed in a biochemical incubator at a temperature of 28 ℃ for an incubation period of 24 h. Afterward, the L1s and unhatched eggs were observed under a microscope and their numbers recorded.

### Fecal egg counts

The McMaster technique was utilized for the quantification of eggs per gram of feces (EPG) in the fecal samples [35]. Fresh fecal samples were collected from the goat’s rectum, and 2 g of feces were precisely weighed and transferred into a small beaker. After mashing the fecal sample, 58 mL of saturated saline solution was added and mixed well, before 1 mL of sample solution was quickly added to the McMaster counting chamber. The chamber was then allowed to settle undisturbed for a duration of 5 min prior to microscopic observation and enumeration. Starting from the 15th day after oral infection with L3s, the excretion of eggs was monitored daily. From the first day of egg detection, a count of eggs was conducted every other day until the end of the experiment (day 64).

### Worm burden

On the 64th day of the experiment, all the goats were slaughtered. The abomasa of goats were ligated at both ends, removed, and cut longitudinally. According to the method used in previous research [34], the *H*. *contortus* adults in the abomasum were counted, and the males and females were distinguished.

### Trehalose content in adult worms

The content of trehalose in different groups of *H*. *contortus* adults was detected by a commercial trehalose content detection kit [36]. Briefly, the standard curve was established with the standard substance, then a 100 mg adult sample was weighed and 1 mL trehalose extract was added, which was placed in a tissue homogenizer to fully break up the sample. After centrifugation at 8000 *g* for 10 min, the supernatant was removed, and the protein concentration in the supernatant was adjusted to be the same. After tenfold dilution of the supernatant, 60 μL of the supernatant sample was mixed with 240 μL of working solution, and the mixture was immersed in water at 95 ℃ for 10 min before naturally cooling to room temperature. Finally, 200 μL was taken into a 96-well plate, placed in a full-wavelength microplate reader, and the absorbance value was measured at OD_620_. According to the standard curve, ΔA determination (*y*, A determination) was put into the formula to calculate the sample concentration (*x*, mg/mL). Calculation formula: fucose content (mg/mg protein) = *x* × sample dilution factor/ sample protein concentration.

### Statistical analysis

GraphPad Premier 6.0 software was used to analyze and plot the data from each group. The results are presented as mean ± standard error of the mean (SEM). Differences between the two groups were determined using a *t*-test, with significance indicated by *P < 0.05, **P < 0.01, ***P < 0.001, and ****P < 0.0001.

## Results

### Polyclonal antibodies against rHcTPS or rHcGOB had no effect on egg hatching

**Fig. 1:**
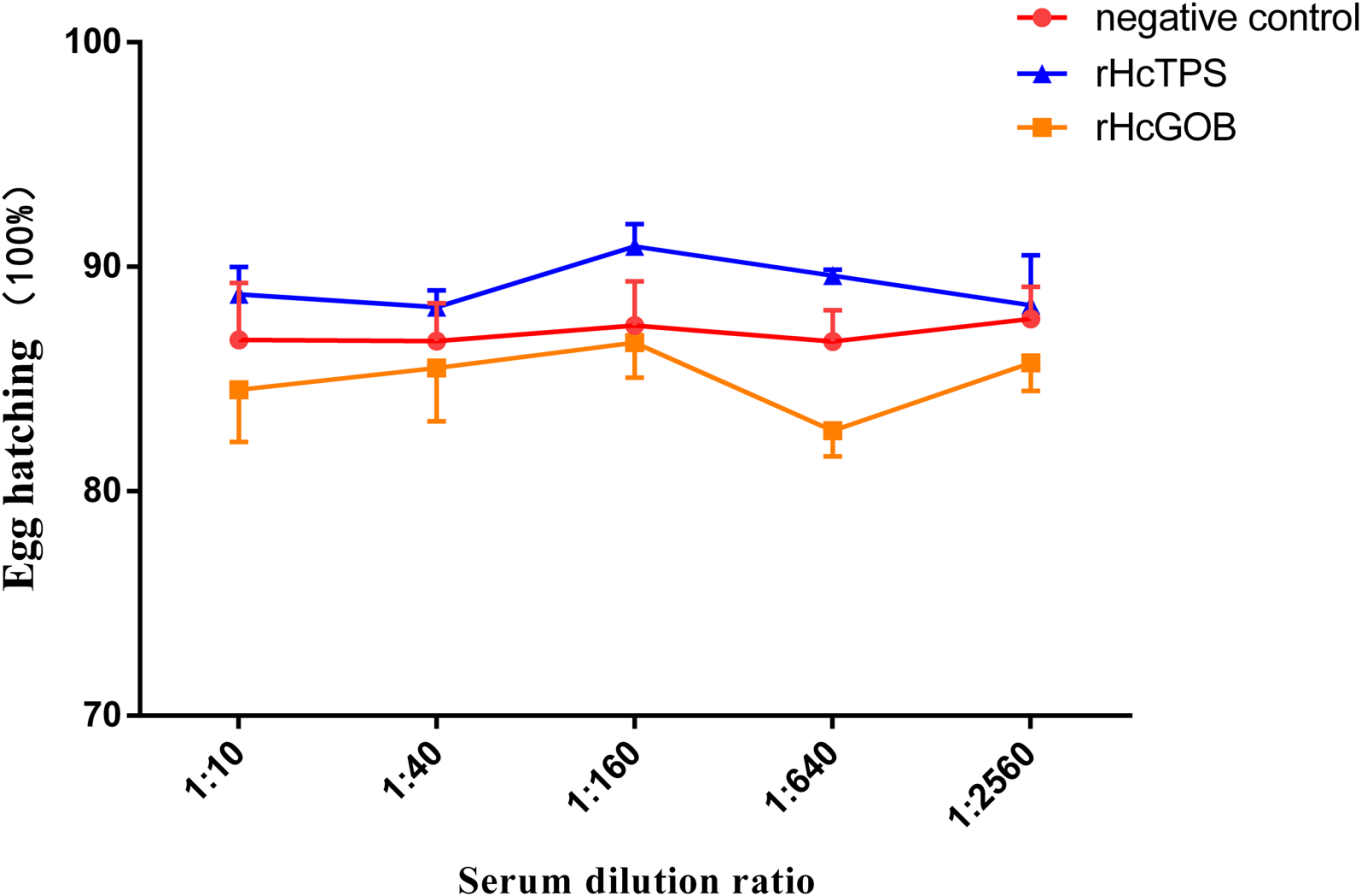
Effect of polyclonal antibodies on the hatchability of eggs. The eggs were incubated with rat serum against recombinant protein and normal rat serum (control) at different dilutions (1:10, 1:40, 1:160, 1:640, and 1:1256) for 24 h, and the hatching rate of eggs was counted. Data are presented as mean ± SEM

The eggs of *H*. *contortus* were incubated with rat serum against rHcTPS protein or rHcGOB protein in different dilutions (1:10, 1:40, 1:160, 1:640, and 1:1256) or normal rat serum (control) for 24 h, and the changes of egg hatching rate were analyzed. At the same time, the blank control group (negative control) and 1 μg/mL albendazole control group (positive control) were set. The results, as shown in Fig. 1, show that the polyclonal antibodies against rHcTPS protein or rHcGOB protein at different dilutions had no significant effect on the hatching rate of eggs.

### Polyclonal antibodies against rHcTPS or rHcGOB inhibited L3 development

**Fig. 2:**
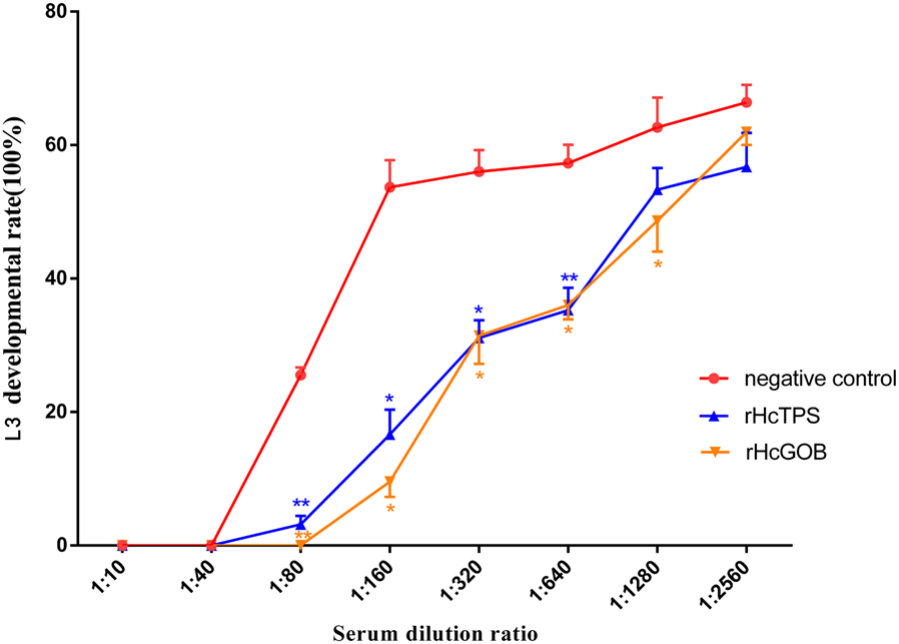
Effect of polyclonal antibodies on L3 development rate. L1s were incubated with polyclonal antibodies against recombinant proteins (rHcTPS protein and rHcGOB protein) at different dilutions (1:10, 1:40, 1:80, 1:160, 1:640, 1:1280, and 1:1256) for 120 h, and the development of larvae was analyzed. Data are presented as mean ± SEM, asterisks indicate significant differences at *P < 0.05 and **P < 0.01 versus the control group

L1s were incubated with polyclonal antibodies against the rHcTPS protein or rHcGOB protein at different dilutions, and the changes in L3 development rate were analyzed. At the same time, the blank control group (negative control) and 1 μg/mL albendazole control group (positive control) were set. Compared with the control group (normal rat serum), the development rate of L3s in the polyclonal antibody group against the rHcTPS protein decreased significantly in the serum dilution ratio of 1:80 to 1:640 (Fig. 2). Compared with the control group (normal rat serum), the development rate of L3s in the polyclonal antibody group against the rHcGOB protein decreased significantly in the serum dilution ratio of 1:80 to 1:1280 (Fig. 2). In addition, the development rate of L3s in the blank control group was 66.7%, and that in the albendazole group was 0%.

### Immunization with the rHcTPS protein or rHcGOB protein elicited specific immune responses in goats

**Fig. 3:**
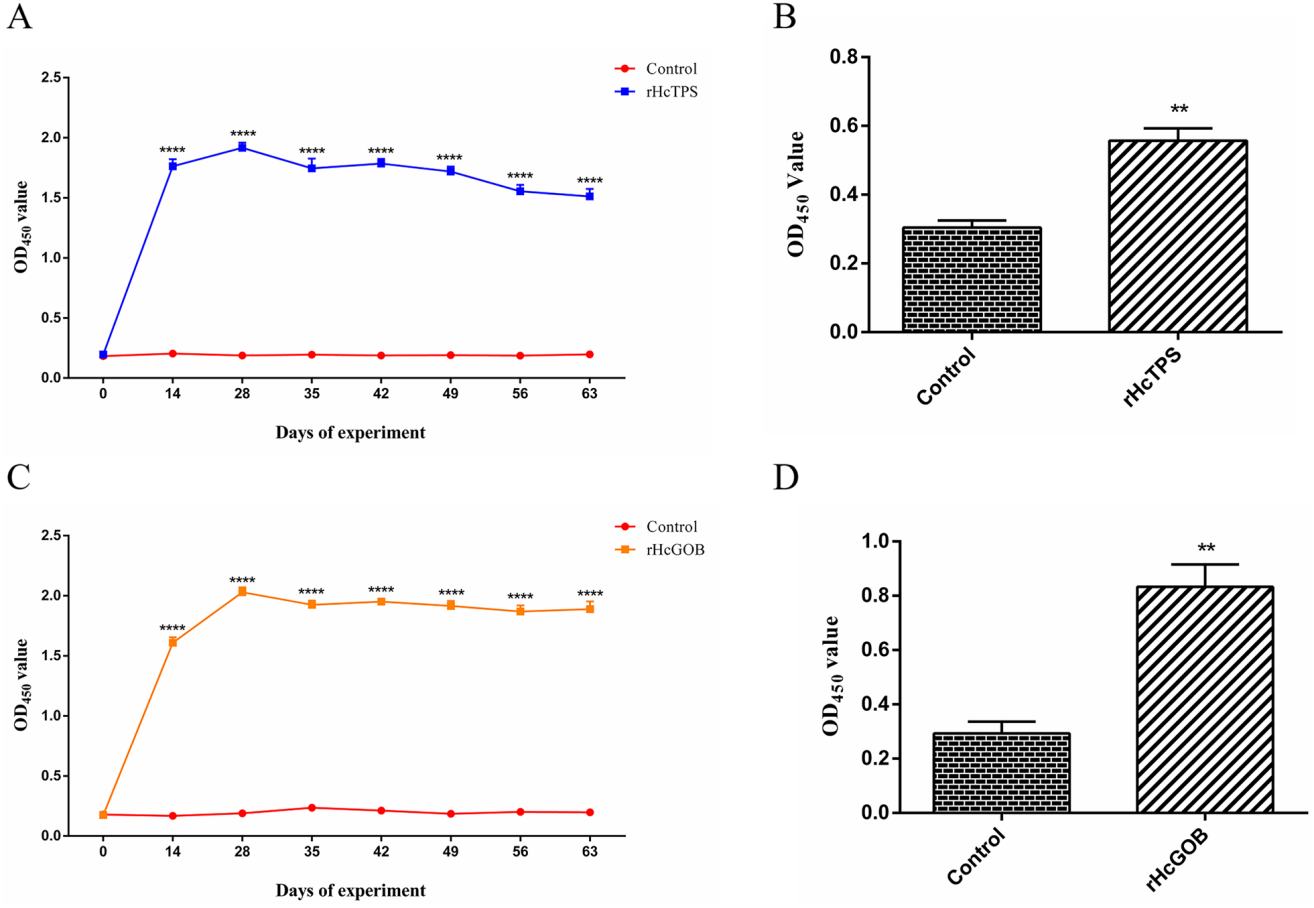
The levels of specific IgG antibodies in the serum and abomasal mucosa of goats were measured. **A** Serum IgG antibodies against rHcTPS protein in the Quin-A adjuvant control group and the rHcTPS protein immunized group were detected by ELISA. **B** Specific IgG antibodies against the rHcTPS protein were detected by ELISA in abomasum mucosa of the Quil-A adjuvant control group and rHcTPS immunized group. **C** Serum IgG antibodies against the rHcGOB protein in the Quin-A adjuvant control group and the rHcGOB protein immunized group were detected by ELISA. **D** Specific IgG antibodies against the rHcGOB protein were detected by ELISA in abomasum mucosa of the Quil-A adjuvant control group and the rHcGOB immunized group. Data are presented as mean ± SEM, asterisks indicate significant differences at **P < 0.01 and ****P < 0.0001 versus the control group

The results are shown in Fig. 3A and C; after the secondary immunization with the rHcTPS protein or rHcGOB protein, the specific immune response in goats was activated, and high levels of specific IgG antibodies were produced in vivo.

From the first immunization to the end of the 64th day of the trial, the specific IgG antibody levels of the rHcTPS protein immunized group and the rHcGOB protein immunized group maintained higher antibody titers compared with the Quil-A adjuvanted control group. However, after L3 infection, the specific IgG antibody level of the rHcTPS protein immunized group and the rHcGOB protein immunized group showed a slight downward trend. Moreover, the specific IgG level in the rumen mucosa of the rHcTPS protein immunized group and the rHcGOB protein immunized group was also significantly increased compared with the Quil-A adjuvant group, indicating that rHcTPS protein and rHcGOB protein activated the local immune response in the rumen (Fig. 3B and D).

### Egg deformity increased in vaccinated groups

**Fig. 4:**
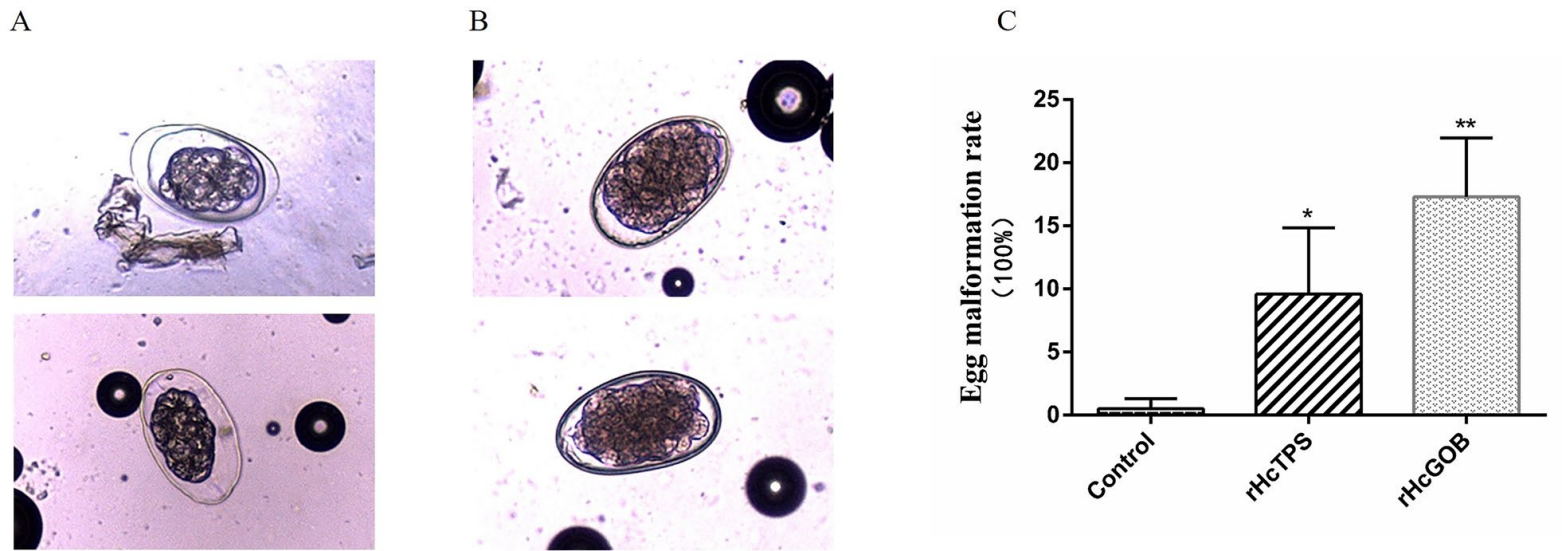
Effect of rHcTPS or rHcGOB protein immunization on the rate of egg malformation. **A** Malformed egg. **B** Normal egg. **C** Statistics of egg malformation rate. Data are presented as mean ± SEM, asterisks indicate significant differences at *P < 0.05 and **P < 0.01 versus the control group

In the EPG counts of the rHcTPS protein immunized group and the rHcGOB immunized group, it was observed that the number of eggs with abnormal morphology and structure (the embryo became smaller and granular, the egg shell became thinner, and the transparent space in the egg shell became larger) was significantly increased (Fig. 4A and B). As shown in Fig. 4C, the egg malformation rates of the rHcTPS protein immunized group and the rHcGOB protein immunized group were 9.59% and 17.30%, respectively, which were significantly different from the control group (Quil-A adjuvant).

### Egg hatching rate decreased in vaccinated groups

**Fig. 5:**
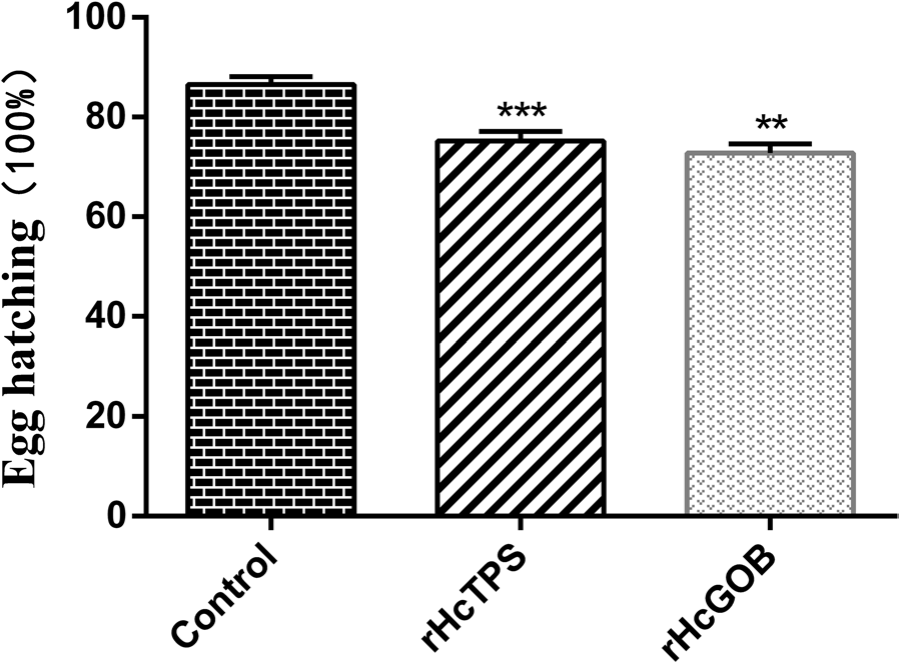
Effect of rHcTPS protein or rHcGOB protein immunization on egg hatchability. The eggs were collected from rectal feces and the purified eggs were incubated at 28 ℃ for 24 h. The egg hatching rate of each immunized group was counted. Data are presented as mean ± SEM, asterisks indicate significant differences at *P < 0.05 and **P < 0.01 versus the control group

As shown in Fig. 5, compared with the control group (Quil-A adjuvant), the hatching rate of eggs in the rHcTPS protein immunized group was significantly reduced by 11.27%, and the hatching rate of eggs in the rHcGOB immunized group was significantly reduced by 13.71%.

### Immunization with rHcTPS protein or rHcGOB protein led to a notable decrease in egg shedding

**Fig. 6:**
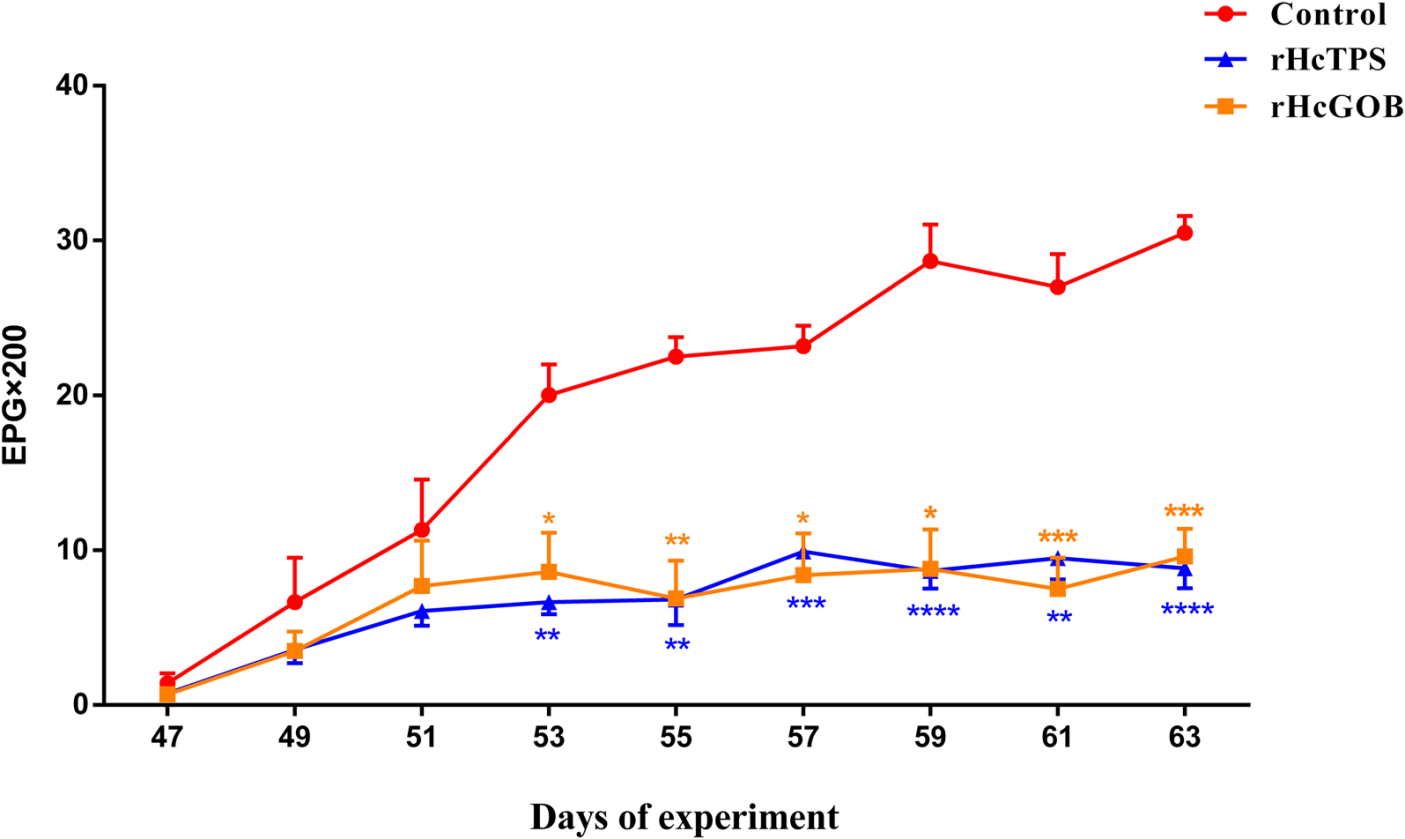
Dynamics of fecal egg counts of different groups. Fresh fecal samples were obtained from the rectum of goats, and the EPG values for each immunized group were quantified using the McMaster method. Data are presented as mean ± SEM, asterisks indicate significant differences at *P < 0.05, **P < 0.01, ***P < 0.001, and ****P < 0.0001 versus the control group

Compared with the control group (Quil-A adjuvant), the EPG values of the rHcTPS protein immunized group and rHcGOB protein immunized group showed a significant reduction on day 53, day 55, day 57, day 59, day 61, and day 63 (Fig. 6). During the entire trial period, the average EPG values of the rHcTPS protein immunized group and rHcGOB protein immunized group decreased by 64.47% and 63.97%, respectively, when compared with the control group (Quil-A adjuvant).

### Worm burden was decreased in goat immunization with rHcTPS or rHcGOB

**Fig. 7:**
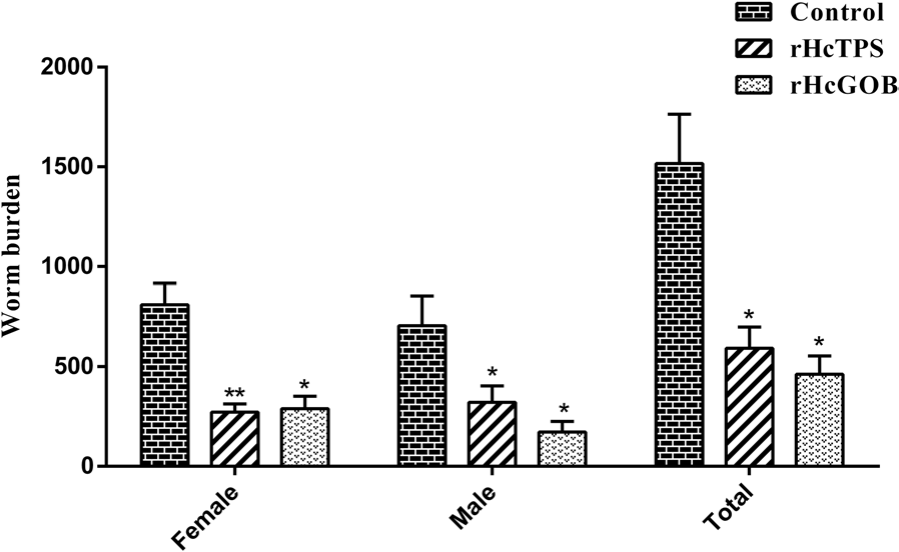
Effect of rHcTPS protein or rHcGOB protein immunization on worm burden. On the 64th day of the experiment (the 35th day post-L3-infection), the enumeration of adult *H*. *contortus* in the abomasum of all goats within each immunized group was conducted, with differentiation between male and female adults. Data are presented as mean ± SEM, asterisks indicate significant differences at *P < 0.05 and **P < 0.001 versus the control group

Compared with the control group (Quil-A adjuvant), the number of adults in the rHcTPS protein immunized group was significantly reduced by 60.93%, including a reduction of 66.53% in female adults and 54.51% in male adults (Fig. 7). Compared with the control group (Quil-A adjuvant), the number of adults in the rHcGOB protein immunized group was significantly reduced by 69.54%, including a reduction of 64.28% in female adults and 75.58% in male adults (Fig. 7).

### Notable reduction in trehalose content in adult worms

**Fig. 8:**
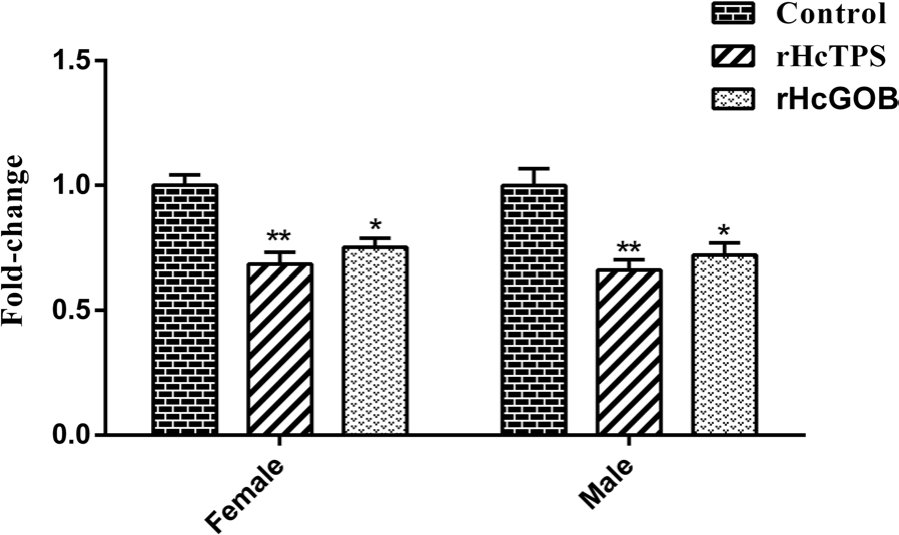
Effect of rHcTPS protein or rHcGOB protein immunization on trehalose content in *H*. *contortus*. The trehalose content in adult *H*. *contortus* (female and male) of different immunized groups was analyzed by commercial trehalose content kit. Data are presented as mean ± SEM, asterisks indicate significant differences at *P < 0.05 and **P < 0.001 versus the control group

Compared with the control group (Quil-A adjuvant), the trehalose content of *H*. *contortus* in the rHcTPS protein immunized group and the rHcGOB protein immunized group exhibited a significant reduction (31.50% and 33.89% for females and males in the rHcTPS protein immunized group, respectively; 24.77% and 27.94% for females and males in the rHcGOB protein immunized group, respectively) (Fig. 8).

## Discussion

Our previous study found that the expression levels of the key enzymes of trehalose synthesis (HcTPS and HcGOB) were high in the egg stage of *H*. *contortus* [25, 26], so we speculated that HcTPS and HcGOB might be related to the development, maturation, hatching, and other life activities of eggs. In addition, chitin is the main component of nematode egg shell and larval sheath membrane, so it is presumed that the enzyme molecules (HcTPS and HcGOB) required for its synthesis play important functions in the process of egg hatching and larval development. Therefore, in this study, polyclonal antibodies of rHcTPS protein or HcGOB protein were co-incubated with eggs in vitro to explore its effect on egg hatching. However, the polyclonal antibodies against rHcTPS protein or rHcGOB protein had no significant effect on the hatching rate of eggs in vitro. There may be two reasons for this: (1) the barrier effect of the egg structure impedes the entry of antibodies and other macromolecular substances, therefore the function of HcTPS and HcGOB in the egg cannot be blocked by antibodies; and (2) the loss of function of HcTPS and HcGOB may indeed have no effect on the hatching of eggs.

The life cycle of *H*. *contortus* includes egg, first-stage larvae (L1s), second-stage larvae (L2s), and infective third-stage larvae (L3s) [37, 38]. The hatching rate of eggs and the development and movement of L3s are frequently utilized as primary screening indicators in anthelmintic drug research [39–41]. This study found that the polyclonal antibodies against rHcTPS protein or rHcGOB protein affected the development process from L1s to L3s in vitro, which may be caused by the blocking of HcTPS or HcGOB in larvae by the polyclonal antibodies; further interfering with the trehalose-chitin synthesis pathway (that is, the development from L1s to L3s may require the participation of substances such as trehalose and chitin). Surprisingly, normal rat serum at 1:40 and 1:80 dilution ratios significantly inhibited the development of L1s to L3s. It is presumed that there may be active substances in rat serum that affect the development of larvae, which may also be one of the reasons why L3s do not infect rats, but the detailed mechanism needs further study.

It is well known that there are five trehalose biosynthesis pathways in prokaryotes, among which, the trehalose-6-phosphate synthase (TPS)/trehalose-6-phosphate phosphatase (TPP) synthesis pathway is the classical pathway of trehalose synthesis, and the only pathway of invertebrate trehalose synthesis [42, 43]. Previous studies have shown that trehalose plays a crucial role in embryonic development, glucose uptake, and resistance to the adverse external environment of helminths [42, 44, 45]. During the process of egg hatching, trehalose serves not only as a stress protector but also as the primary energy source, capable of being catabolized to provide glucose and energy [46–48]. In this investigation, the administration of a rHcTPS protein vaccine and a rHcGOB protein vaccine resulted in aberrant egg phenotypes, accompanied by a reduction in egg hatching rates of 11.27% and 13.71%, respectively. These findings are consistent with previous reports on *Brugia malayi* [42] and contribute to our understanding of the impact of these vaccines on parasite development. Chitin is widely recognized as a crucial component of nematode egg shells, serving a pivotal role in preserving the morphology and functionality of the eggs [49–51]. Moreover, this study revealed that both rHcTPS protein and rHcGOB protein immunization resulted in a significant decrease in trehalose content in *H*. *contortus*. This suggests that rHcTPS protein and rHcGOB protein immunization interfered with the trehalose-chitin synthesis pathway, which may be the reason for egg malformation and reduced egg hatching rate. Previous studies have shown that the loss of TPP activity or TPP gene silencing in *Caenorhabditis elegans* can lead to the accumulation of high concentrations of intermediate product T6P in vivo, which in turn leads to larval lethality [52]. Compared with the group immunized with rHcTPS protein, the group immunized with rHcGOB protein exhibited a higher incidence of egg malformations and lower egg hatching. This may be attributed to the inhibition of HcGOB enzyme activity in the rHcGOB protein-immunized group by specific antibodies, leading to an accumulation of the intermediate product, T6P, and subsequent toxic effects. Furthermore, this study is the first to report that immunization with recombinant protein vaccines resulted in an increase in deformed eggs and a decrease in egg hatching rate, which may be more beneficial for the prevention and control of hemonchosis. Therefore, it is necessary to further explore the detailed mechanisms of the rHcTPS protein vaccine and the rHcGOB protein vaccine causing abnormal phenotypes and reduced hatching rates of eggs in subsequent studies.

Worm reduction rate and egg reduction rate are the most important indicators in evaluating the effects of worm vaccines [53]. Previous studies have demonstrated that the downregulation of the TPP gene in infected *B. malayi* larvae, using siRNA, resulted in an 84.9% reduction in adult worm burden following intraperitoneal infection of the host [42]. Moreover, recombinant TPP protein vaccination provided significant protection against *B*. *malayi* infection, reducing the worm burden by 67.8%. In this study, the rHcTPS protein and rHcGOB protein immunized groups exhibited a significant reduction in the number of eggs and adults compared with the Quil-A adjuvant control group. Specifically, there was a 64.47% and 63.97% decrease in average EPG, as well as a 60.93% and 69.54% decrease in the average number of adults for the rHcTPS protein immunized group and rHcGOB protein immunized group, respectively. These findings suggest that immunization with rHcTPS protein and rHcGOB protein can induce substantial protective effects against *H*. *contortus* infection. The mechanism of rHcTPS protein and rHcGOB protein immune protection against *H*. *contortus* infection may be as follows: first, immunization with rHcTPS protein and rHcGOB protein can stimulate the host to produce high levels of specific antibodies. During the blood-feeding process of *H*. *contortus*, these circulating antibodies enter the parasite’s digestive tract and specifically target the native HcTPS protein and HcGOB protein located on its surface. Our previous study revealed a similar distribution pattern of HcTPS and HcGOB in adult *H*. *contortus*, predominantly localized to the intestinal microvilli [25, 26]. This localization bears resemblance to that of Barbervax^®^ vaccine antigens, thereby disrupting the synthesis pathway of trehalose-chitin (impacting trehalose and chitin synthesis) and consequently impeding the growth, development, and colonization of *H*. *contortus* in the abomasum. Second, the subunit vaccines of rHcTPS protein and rHcGOB protein stimulate the production of specific antibodies in the body. Upon infection with *H*. *contortus*, these specific antibodies can effectively block immunosuppressive molecules (our previous investigation demonstrated that HcTPS and HcGOB function as immunosuppressive molecules, capable of suppressing the Th2 immune response in the host [25, 26]), reactivate the host immune system, and subsequently initiate an immune response against the *H*. *contortus*.

The immunosuppressive molecules of *H*. *contortus* have been demonstrated in previous studies to be a promising group of vaccine candidates [14, 34, 54]. Tian et al. discovered that the transthyretin domain containing protein (HcTTR) functions as an antagonist of interleukin-4 (IL-4) [55]. Furthermore, the rHcTTR protein vaccine demonstrated a significant immune-protective effect against *H*. *contortus* infection in goats, resulting in a 66.4% reduction in adults and a 63.7% decrease in eggs per gram (EPG) [34]. Similarly, Lu et al. discovered that the hydrolase structure-containing protein (HcABHD) could effectively inhibit the secretion of IL-4, interferon-γ (IFN-γ), transforming growth factor beta (TGF-β), and other cytokines in PBMCs [56]. Furthermore, immunization with the rHcABHD protein vaccine demonstrated a significant protective effect on goats against *H*. *contortus* infection, resulting in a 74.2% reduction in the number of adults and a 54.0% decrease in EPG levels [53]. Recent research has demonstrated that the 14-3-3 protein (rHcftt-2) functions as an inhibitory molecule of PBMCs [57]. Furthermore, immunization with the rHcftt-2 protein vaccine has been shown to confer a certain level of protection against *H*. *contortus* infection in goats, resulting in a reduction of 32.33% and 26.46% in the number of adults and EPG, respectively [54]. The findings of this study align with the aforementioned results, demonstrating that both the rHcTPS protein vaccine and rHcGOB protein vaccine confer robust immune protection in goats against *H*. *contortus* infection. Furthermore, the rHcTPS protein vaccine and rHcGOB protein vaccine not only demonstrated significant efficacy in reducing the population of eggs and adults but also exerted an impact on the progeny of the remaining *H*. *contortus* (resulting in a substantial increase in the number of deformed eggs). Hence, it is imperative to conduct a subsequent investigation aimed at monitoring the phenotypic alterations in larvae, adults, and eggs following the silencing of the HcTPS gene and HcGOB gene using siRNA or inhibitors. This will facilitate a comprehensive understanding of the roles played by HcTPS and HcGOB molecules across various developmental stages of *H*. *contortus*.

It is well-known that nematodes possess a complex life cycle, encompassing several distinct developmental stages (such as eggs, larvae, and adults), with each stage expressing diverse antigens that exhibit a high degree of heterogeneity [58, 59]. These antigens might also exhibit variations among nematode populations in diverse hosts and geographical regions, thereby posing an extremely formidable challenge in screening for a single vaccine antigen capable of inducing robust and long-lasting immune protection. Furthermore, diverse immune protocols, immune dosages, and adjuvants also exert an influence on the immune efficacy of vaccine antigens. Regrettably, this study has not yet accomplished the exploration of the effects of different adjuvants, immune doses, and immune programs on the immune efficacy of recombinant protein (rHcTPS and rHcGOB) vaccines. Several vaccine trials have been conducted for over three decades in the pursuit of effective recombinant protein vaccine development against *H*. *contortus*, and despite employing multiple approaches to test numerous antigens, only partial immunity is produced [60]. It is intriguing that Barbervax^®^, the sole commercially available vaccine for hemonchosis, employs a native protein antigen as its vaccine antigen, effectively facilitating the host’s resistance to infection by *H*. *contortus* (https://barbervax.com/about/). The conformational disparities between recombinant protein antigens (rHcTPS and rHcGOB) and native nematode antigens might also be one of the factors influencing the efficacy of recombinant vaccines. Consequently, further research is required to investigate the potential of extracting native HcTPS protein and HcGOB protein from *H*. *contortus* as vaccine antigens.

## Conclusions

Vaccination with rHcTPS and rHcGOB resulted in a remarkable reduction in worm burden and egg shedding. Furthermore, an increased aberrant phenotype of eggs and decreased egg hatching rates were also found. These findings demonstrate the immune protective effects of HcTPS and HcGOB against *H*. *contortus* infection. The results indicate that enzymes involved in trehalose-chitin synthesis could be promising candidates for vaccine development.

## Data Availability

All data generated or analyzed during this study are included within the article.

## Abbreviations

HcTPS: Trehalose-6-phosphate synthase of *Haemonchus contortus*
HcGOB: Trehalose-6-phosphate phosphatase of *Haemonchus contortus*
*H*. *contortus*: *Haemonchus contortus*
L1s: First-stage larvae
L2s: Second-stage larvae
L3s: Infective third-stage larvae
EPG: The quantification of eggs per gram of feces
rHcTPS: Recombinant HcTPS
rHcGOB: Recombinant HcGOB
PBS: Phosphate-buffered saline
IL: Interleukin
PBMC: Peripheral blood mononuclear cell
